# Advancing cardiac care through national registries

**DOI:** 10.1007/s12471-024-01878-4

**Published:** 2024-05-31

**Authors:** Pim van der Harst

**Affiliations:** Department of Cardiology, Division of Heart and Lungs, University Medical Centre Utrecht, Utrecht, The Netherlands

In this issue of the *Netherlands Heart Journal*, the critical role of registries in advancing cardiac care is highlighted by two registry-focused articles and a promising single-centre study.

As detailed by Derks et al., the Netherlands Heart Registration (NHR) now encompasses over 1.5 million procedures and data from 75 centres [1]. This extensive coverage of key cardiac procedures makes the NHR a cornerstone in the quality assessment efforts of the Netherlands Society of Cardiology (NVVC) and the Dutch Association for Thoracic Surgery (NVT), demonstrating the powerful role of collaborative data collection by cardiologists and cardiothoracic surgeons and their analysis to advance real-world clinical practice and patient care protocols.

The Dutch Idiopathic Ventricular Fibrillation Registry, presented by Verheul et al. and including 11 contributing hospitals, showcases the benefits of specialised and unique registries in enhancing diagnostics and understanding cardiac conditions [2]. Indeed, De Groot, in his commentary, briefly points out that we should continue to aim to reduce the incidence of VF deemded to be idiopathic by furthering our phenotyping efforts and underscoring future research opportunities to reveal substrates to refine our diagnostic approaches [3].

The study on ECMELLA treatment by Balder et al., while currently limited to a single centre, emphasises the value of detailed clinical data in evaluating new cardiac support technologies [4]. The potential for expanding this into a national registry could significantly enhance our understanding of treatment efficacy and the safety of this costly procedure.

Ultimately, registries and quality data play vital roles not only in our clinical settings but also in informing government decision-makers on appropriate care and reimbursement. The active involvement of physicians in data collection and analysis is essential. We encourage all readers to actively engage in these efforts and to generously share their findings.
